# Pharmacological effects of monoterpene carveol on the neuromuscular system of nematodes and mammals

**DOI:** 10.3389/fphar.2024.1326779

**Published:** 2024-01-22

**Authors:** Maja Stojković, Zoran Todorović, Dragana Protić, Strahinja Stevanovic, Dragana Medić, Claude L. Charvet, Elise Courtot, Djordje S. Marjanović, Jelena Nedeljković Trailović, Saša M. Trailović

**Affiliations:** ^1^ Department of Pharmacology, Clinical Pharmacology and Toxicology, Faculty of Medicine, University of Belgrade, Belgrade, Serbia; ^2^ PR CYNNAB, Belgrade, Serbia; ^3^ Department of Pharmacology and Toxicology, Faculty of Veterinary Medicine, University of Belgrade, Belgrade, Serbia; ^4^ INRAE, Université de Tours, ISPF-37380, Nouzilly, France; ^5^ Department of Nutrition and Botany, Faculty of Veterinary Medicine, University of Belgrade, Belgrade, Serbia

**Keywords:** carveol, *C. elegans*, *Xenopus* oocytes, *A. suum*, AChR

## Abstract

The control of parasitic nematode infections relies mostly on anthelmintics. The potential pharmacotherapeutic application of phytochemicals, in order to overcome parasite resistance and enhance the effect of existing drugs, is becoming increasingly important. The antinematodal effects of carveol was tested on the free-living nematode *Caenorhabditis elegans* and the neuromuscular preparation of the parasitic nematode *Ascaris suum*. Carveol caused spastic paralysis in *C*. *elegans*. In *A. suum* carveol potentiated contractions induced by acetylcholine (ACh) and this effect was confirmed with two-electrode voltage-clamp electrophysiology on the *A*. *suum* nicotinic ACh receptor expressed in *Xenopus* oocytes. However, potentiating effect of carveol on ACh-induced contractions was partially sensitive to atropine, indicates a dominant nicotine effect but also the involvement of some muscarinic structures. The effects of carveol on the neuromuscular system of mammals are also specific. In micromolar concentrations, carveol acts as a non-competitive ACh antagonist on ileum contractions. Unlike atropine, it does not change the EC_50_ of ACh, but reduces the amplitude of contractions. Carveol caused an increase in Electrical Field Stimulation-evoked contractions of the isolated rat diaphragm, but at higher concentrations it caused an inhibition. Also, carveol neutralized the mecamylamine-induced tetanic fade, indicating a possibly different pre- and post-synaptic action at the neuromuscular junction.

## 1 Introduction

Parasitic nematode infections are widespread in nature, affecting humans as well as animals. The most common nematodes in humans and animals are ascarids, pinworms, trichinella, and hookworm. Control of these infections relies mostly on chemotherapeutics (anthelmintics), but resistance has developed against most of these broad-spectrum drugs in many parasite species. The problem is that after the introduction of avermectins, most of the newly discovered substances did not meet the requirements to become potentially effective and safe antinematodal drugs. On the other hand, a significant level of resistance to existing anthelmintics requires special attention and indicates the need for new drugs or new protocols in order to improve the effectiveness of existing drugs without increasing doses. Continued use of existing anthelmintics will proceed to increase the level of resistance. We already know well that cure rates are now often less than 100%, and parasite resistance to agents that act on the neuromuscular systems is present in a wide range of animal and human host parasites (Martin and Robertson, 2010).

There is a need to focus research on drug discovery and development to overcome the issue of increasing resistance and reduced efficacy against existing drugs. We are particularly interested in phytochemicals - compounds that occur naturally in plants as secondary metabolites. Plants contain products from primary and secondary metabolisms that are of great importance for their existence. The secondary metabolism of plants contributes towards their colonization of terrestrial environments (Bakkali et al., 2008; Poser and Mentz, 2010; Sharifi-Rad et al., 2019). One of the principal fractions of chemical substances found in plants are essential oils (EOs). EOs consist primarily of a mixture of volatile hydrophobic secondary metabolites with marked odors and great commercial value. In plants, EOs act as protective agents against predators and attractants for pollinators (Craveiro and Machado, 1986; Simões et al., 2010). The main chemical compounds found in EOs are terpenoids and phenylpropanoids (Benelli, 2015; Benelli and Mehlhorn, 2016) that are widely used as bioactive molecules in biology, agronomy, medicine, and pharmaceutical sciences. Among the medical-pharmaceutical activities of these compounds are antitumor, anthelmintic, and larvicidal (Silva-Santos et al., 2006; Sousa et al., 2007). In the previous research, we have shown that monoterpenoid carvacrol acts on the neuromuscular system of nematodes and exhibits an inhibitory effect on contractions caused by acetylcholine (ACh) (Trailović et al., 2015). In our following study, carvacrol dominantly exhibited characteristics of a non-competitive antagonist of nicotinic acetylcholine receptor (nAChR) in *Ascaris suum* (*A. suum*), and enhances the inhibitory effect of monepantel (Marjanović et al., 2021; Trailovic et al., 2021).

In current study we decided to examine in more detail the potential antinematodal effects of carveol a constituent of spearmint EO. The monoterpene carveol is a molecule widely used in the cosmetic industries (Bhatia et al., 2008). Carveol has also been shown to be an interesting compound with pharmacological activities, which include a repellent effect when used against the African malaria mosquito, *Anopheles gambiae* (Omolo et al., 2004), a nematocidal action against the root-knot nematode *Meloidogyne incognita* (Echeverrigaray et al., 2010), and antibacterial activity (Guimarães et al., 2019). Carveol has also shown a myorelaxant effect in isolated rat aorta sections in smooth muscle studies (Cardoso-Teixeira et al., 2018). The purpose of the presented research is to determine the anthelmintic potential of carveol and compare its effects on the neuromuscular system of nematodes with the effects on the smooth and skeletal muscles of mammals (a potential host). These data can be used for the potential antiparasitic application of carveol, alone or in combination with existing antiparasitic drugs.

The aims of present study were: (i) to make *in silico* predictions of the binding of carveol to the nAChR in *A. suum* and compare it with the binding properties of two other monoterpenes, carvacrol and geraniol; (ii) to test effects of carveol on *C. elegans*; (iii) to investigate action of carveol on the model of the neuromuscular preparation of *A. suum*, (iv) to investigate effects of carveol on *A. suum* nAChR expressed in *Xenopus* oocytes. Finally (v), this study aimed to compare carveol effects obtained in nematodes and mammals (isolated rat ileum and diaphragm).

## 2 Materials and methods

### 2.1 Docking testing

The *in silico* structure-based docking method was used to measure the affinity of the ligand to the receptor by the smina program (version of AutoDock Vina) (http://pubs.acs.org/doi/abs/10.1021/ci300604z). The sklearn library and the Mini-Batch-Kmeans algorithm (Pedregosa et al., 2011) were used for statistical processing. The model of nAChR, ACR16 receptor of *A. suum* (see uniprot id from the Supplementary Figure S1) (The UniProt Consortium, 2023), was obtained by the Phyre2 service. Docking is limited to the subunit of the receptor with two domains, alpha and beta (Supplementary Figure S1) (Unwin, 2005; https://www.rcsb.org/structure/2BG9). Carveol XLogP3 2.1 (cyclohexene 2.9), carvacrol XLogP3 3.1 (benzene 2.1), and geraniol XLogP3 2.9 were chosen as ligands.

### 2.2 Investigation on *C. elegans*

*C. elegans*, N2 wild-type is obtained from the *Caenorhabditis* Genetics Center (Charvet et al., 2018). Nematode Growth Medium was used for the cultivation of *C. elegans* (NGM, US Biological, Life Sciences, United States). After culturing on the NGM plates for 6 days, the worms were collected by cutting the medium into 3 × 3 mm squares and transferred to glass tubes with 2 mL of 0.1M NaCl. The tubes were placed in a shaker until complete dissolution of the substrate and release of the worms. The tubes were then centrifuged for 4 min at 2,500 r/min, and 400 μL of supernatant were transferred to a second test tube. This was followed by another minute of centrifugation, at 2000 r/min. After that, 20 μL of suspension of nematodes were inoculated on the Petri dish (diameter 3 cm) with 2.5 mL of NGM substrate and increasing concentrations of carveol (1, 3, 10, 30, 100, 300μM, 1 and 3 mM), The titer of adult worms was 21–35/20 μL and each concentration was tested on three Petri dishes. The three Petri dishes without the added test substances were untreated controls.

The plates inoculated with *C. elegans* were placed in a thermostat (Memmert IN30, Germany) at 20°C for 24 and 48 h. After incubation, the plates were observed under an inverted microscope (Motic AE 31, PRC) and the movement and pharyngeal pumping of *C. elegans* were recorded with a camera (Motic 5 MP, NRK) on the hard disk of a PC, for later analysis. The survival rate of the adult *C. elegans* was determined in the medium with carveol as well as in the untreated control medium. The lethality was determined by the cessation of movement and pharyngeal pumping. *C. elegans* was considered dead when it did not move and did not respond to repeated touching with a probe. Mortality was calculated for each treatment after 24 and 48 h and expressed in percentages.

### 2.3 Investigation on *A*. *suum* (Nematode muscle contraction assay)

Adult female *A. suum* were collected weekly from the slaughterhouse Ambar (Surčin, Serbia). After collection, the worms were placed and maintained in Locke’s solution of the following content (mM): NaCl 155, KCl 5, CaCl_2_ 2, NaHCO_3_ 1.5 and glucose 5. Temperature was maintained at 32°C–34°C using a water bath and Locke’s solution was changed twice a day. Each batch of worms was used for experiments within 4 days of collection.

Ascaris muscle flaps for contraction studies were prepared as described in Trailović et al. (2015). The preparations were allowed to equilibrate for 15 min under an initial tension of 0.5 g. Contractions were monitored after increasing concentrations of acetylcholine (1, 3, 10, 30 and 100 μM) and then in the presence of carveol or carveol and atropine. The maximum contraction was observed prior to washing and subsequent application of ACh, ACh and carveol, ACh, carvacrol and atropine. The interval between the application of increasing doses of ACh was 1 min and 2 min when the preparation was incubated with carveol or carveol with atropine. The responses for each concentration were expressed in grams (g), produced by each individual flap preparation. Contractions were monitored and recorded in real time on a PC computer, using a BioSmart interface, and eLAB 44 software (ElUnit, Belgrade). Sigmoidal concentration-response curves for ACh effects in the absence or presence of carveol/atropine were described by the Hill equation.

### 2.4 Electrophysiological studies of *A*. *suum* nAChR in *Xenopus laevis* oocytes

The functional expression of *A. suum* ACR-16 nAChR in *Xenopus laevis* oocytes was carried out as described previously (Abongwa et al., 2016). Briefly, defolliculated *Xenopus laevis* oocytes (Ecocyte Bioscience) were micro-injected with 36 nL of Asu-ACR-16 cRNA at 50 ng/μL using the Nanoject II microinjector (Drummond). Two micro-electrode voltage-clamp experiments were performed 3 days after micro-injections as previously described (Trailović et al., 2021). Data were collected and analyzed using the pCLAMP 10.4 package (Molecular Devices).

### 2.5 The investigation on rat isolated ileum and diaphragm (Mammalian skeletal and smooth muscle contraction assay)

White male Wistar rats, weighing 150–200 g, were housed under standard conditions for laboratory animals in groups of four in home cages (42.5 × 27 × 19 cm) under standard conditions: temperature of 21°C ± 1°C, relative humidity of 55%–60% and 12/12 h light/dark cycle. Food and water were provided *ad libitum*, except during experimental procedures. All procedures in the study conformed to EEC Directive 86/609 and were approved by the Ethics Committee of the Faculty of Veterinary Medicine, University of Belgrade.

Male Wistar rats were anesthetized with thiopentone and decapitated. Thiopentone sodium (PANPHARMA, France) was administered using an intravenous cannula (OptivaTM 2, Johnson & Johnson, Arlington, TX, United States) via the lateral tail vein at a dose of 35 mg/kg body weight. Diaphragm strips for contraction studies were prepared as described in Trailović et al. (2015). Contractions were monitored after Electrical Field Stimulation (EFS). The pair of platinum electrodes were placed parallel to the muscles and EFS was performed by application of tetanic pulses (10 μs widths, 50 Hz frequency, 30 V voltage, and 2 s duration) in trains of five pulses every 30 s, with a resting period of 3 min between trains. EFS was generated by the BioSmart digital stimulator with software control (El Unit, Belgrade). Isometric muscle force production was determined after 30 min of equilibration time (control). The amplified signals were recorded online by an eLAB 44 software (ElUnit, Belgrade). To confirm that the electrical field stimulation technique induced muscle contraction only via the neuromuscular end-plate, the nicotinic antagonist mecamylamine (100 μM) was used (Papke et al., 2001). The effect of carveol (10, 30, 100 and 300 μM) on contractions of the isolated diaphragm caused by EFS was tested. Tissues were washed each time after recording effects of carveol or mecamylamine.

Ileum segments were prepared for testing contractions as described in (Trailović et al., 2017). After 30 min of the equilibration period, until a stable baseline was attained, increasing concentrations of ACh (1, 3, 10, 30 and 100 μM) were administrated and the maximum contraction was observed before washing and subsequent application of the next concentration of ACh. Afterwards the contractile effect of the same concentrations of ACh was tested in the presence of 100, 300 and 1,000 μM of carveol or 3, 10, 30 and 100 nM of atropine.

In a separate series of tests, ileum preparations were subjected to EFS by packages containing 5 stimulations every 60 s (EFS: 50 Hz, for 2 s, 1.0 ms pulse duration, 50 V intensity), with resting periods of 5 min between each package. EFS was generated by the digital stimulator BIOSMART 150 with software control (El Unit, Belgrade), while EFS was delivered by a pair of platinum electrodes, placed parallelly to the preparation. After two series of stable contractions, the response was observed in the presence of carveol. The amplified signals have been recorded online by SMARTPLUS 150 software (El Unit, Belgrade).

### 2.6 Drugs

Acetylcholine, atropine, mecamylamine and carveol were obtained from Sigma-Aldrich Co. (St Louis, MO, United States). Acetylcholine, mecamylamine, and atropine were dissolved in the APF-Ringer and Tyrode solution. Carveol, was dissolved in ethanol, with a final concentration of ethanol in the APF-Ringer and Tyrode Solution of 0.1 %v/v.

### 2.7 Statistical analyses

The results of muscle contraction assay are expressed as means ± S.E. in grams (g) of contractions. Sigmoid concentration dose-responses were described by the equation as follows: % response = 1/1 + [EC_50_/Xa]nH, where EC_50_ is the concentration of the agonist (Xa) producing 50% of the maximum response and nH is the Hill coefficient (slope). Prism 6.0 (GraphPad Software, San Diego, CA.) was used to estimate the constants EC_50_ and nH, by non-linear regression for each preparation. We determined the mean contraction responses to each concentration of ACh (control dose-response: CR[ACh]) and compared with responses in presence of carveol. Other data was analyzed by ANOVA using Prism 6.0, and the differences were considered significant when the *p*-value is <0.05.

## 3 Results

### 3.1 Molecular docking analyses

**FIGURE 1:**
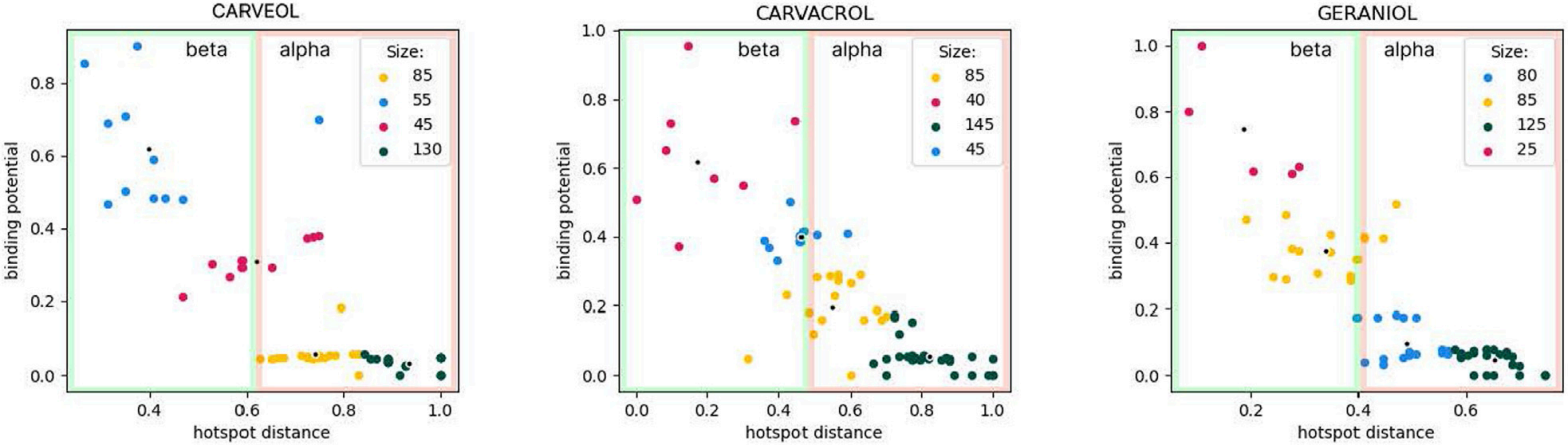
Cluster analysis using K-means. Size indicates the number of molecule binding poses hitting the specific binding potential energy range (normalized kcal/mol) and color-coded using red-to-green scheme (red—least number of hits; green—most abundant hits). Carveol: blue cluster hits are orthosteric; Carvacrol: red/blue clusters hits are allosteric; Geraniol: red cluster holds orthosteric hits, yellow-hits spanning both extracellular and transmembrane domains.

In molecular docking analyses we used the model of homomeric *A*. *suum* nAChR, ACR16 (uniprot: A0A0K0QSL7_ASCSU). The model contains an alpha and a beta domain, namely: alpha domain (alpha subunit, containing 7 alpha helices, alpha 7 domain, transmembrane) and beta domain (beta subunit, extracellular domain containing loops B and C, outer vestibule) as described in PDB entry 2BG9, nicotinic aceylcholine receptor *T. marmarota* at 4Å resolution (Unwin, 2005). The tested ligands were carveol, carvacrol and geraniol, and the common property for all ligands is diffusion through the membrane. Therefore, the affinity at several places in the alpha domain for each of the ligands must not be ignored. However, carveol and carvacrol probably participate in the activation mechanism in the beta domain. Only the organization of interactions of geraniol in the alpha domain shows distinctive binding patterns. Furthermore, the differences are indicated by the lower affinity of geraniol for the receptor. On the other hand, unique to carvacrol is the possibility of pi-stacking and cation-pi interactions due to benzene in the structure. This may explain the different affinities compared to carveol in the beta domain. Carvacrol shows affinity for an allosteric binding site in the beta domain (a possible allosteric site composed of two sub-sites located close to each other). Geraniol potentially binds to the receptor at two sites in the alpha domain with lower affinity than carveol and carvacrol. While, carveol has an affinity for two binding sites that are distant, i.e. one in the beta and one in the alpha domain. The geometry of the binding sites is different from those of carvacrol and geraniol. In the beta domain, carveol shows the highest affinity for the orthosteric binding site (Figure 1).

### 3.2 Investigation on *C. elegans*

To verify the results of docking testing of the cholinergic properties of carveol we examined its effect on the free-living nematode *C. elegans*. The Median Lethal Concentration (LC_50_) of carveol after 24 h of exposure was 490.10 ± 1.40 μM, and it did not change significantly after 48 h of exposure (430.50 ± 1.35 μM).

By analyzing real-time motility recordings, we noticed that exposure to carveol resulted in spastic paralysis. Also, when carveol was in the medium, pharyngeal pumping stopped first (Supplementary Figure S2), while the worms still responded by moving to the touch stimulus.

### 3.3 Investigation of *A*. *suum* muscle contractions

**FIGURE 2:**
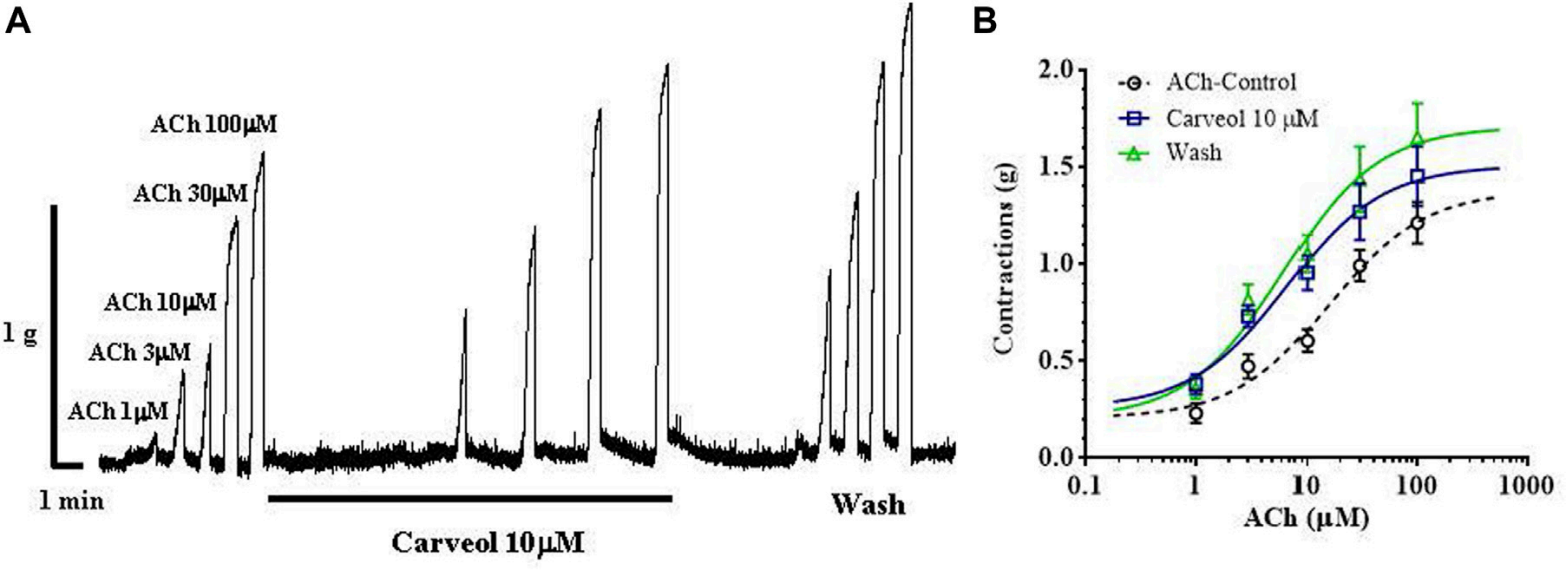
**(A)** Original recording of isometric contractions of *A. suum* muscle flap induced by increasing acetylcholine concentrations and the effect of carveol (on those contractions); **(B)** The concentration-response plot (non-linear regression) for ACh control (*n* = 6), in the presence of carveol 10 µM and after washing (n5) (mean ± S.E).

We checked the results obtained on the free-living nematode *C. elegans* on the contraction model of the neuromuscular preparation of the parasitic nematode *A. suum* (Figure 2A). Initially, we tested the effects of 1, 3 and 10 μM of carveol on ACh-induced contractions. Carveol concentrations of 1 and 3 μM had no or very weak effect on the amplitude of contractions and the EC_50_ value of acetylcholine. Therefore, we continued testing with 10 μM of carveol. The EC_50_ value of ACh for control contractions was 15.97 ± 1.56 μM, while in the presence of carveol 10μM, the EC_50_ was 6.63 ± 1.82μM, which is a significant decrease (*p* = 0.0008). After washing, the EC_50_ of ACh was further significantly reduced to 6.14 ± 1.80 μM (*p* = 0.0004) (Figure 2B). At the same time, carveol increased the maximal contractile response (E_max_) to ACh from the control 1.37 ± 013 g to 1.52 ± 0.12 g. The E_max_ continued to increase after washing and reached 1.72 ± 0.14 g (Figures 2A, B).

**FIGURE 3:**
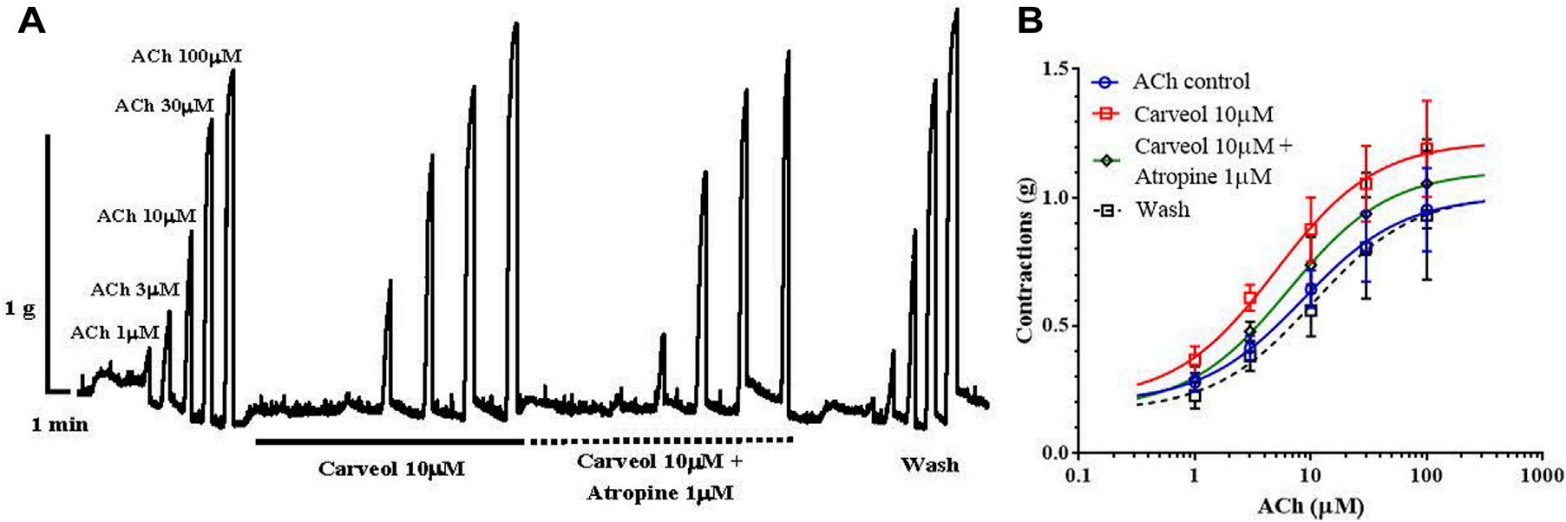
**(A)** Original recording of isometric contractions of *A. suum* muscle flap induced by increasing acetylcholine concentrations and the effect of carveol and carveol and atropine (on those contractions); **(B)** The concentration-response plot (non-linear regression) for ACh control (*n* = 6), in the presence of carveol 10µM, combination carveol 10 µM + atropine 1 µM and after washing (*n* = 6) (mean ± S.E).

It was further interesting to check whether this potentiating effect of carveol on ACh-evoked contractions is sensitive to atropine (Figure 3A). The EC_50_ value for ACh in the control series of contractions was 8.52 ± 2.34 μM, while in the presence of carveol (10 μM) it was reduced by more than 40% (but not significant, *p* = 0.791) to 4.99 ± 2.33 μM. Atropine 1 μM insignificantly increased the EC_50_ of ACh to 5.76 ± 2.63 μM (*p* = 0.887), but after removal of carveol and atropine, the EC_50_ value was slightly higher than the control value, reaching 10.52 ± 3.31 μM (Figure 3B). As in the previous study, carveol increased the E_max_ value of ACh-evoked contractions from the control 1.01 ± 0.13g to 1.22 ± 0.13 g (for 20%), while in the presence of atropine E_max_ decreased to 1.11 ± 0.13 g. After washing, the E_max_ of contractions was 1.02 + 0.21 g, which corresponds to the control value. However, these differences did not reach the level of significance. Apparently, the effect of atropine is reversible, as we observed in previous studies with the neuropeptide AF2 (Trailovic and all, 2005) and the muscarinic agonist 5-methylfurmethiodide (Trailovic and all, 2008). Unfortunately, there is still not much data on the more detailed characteristics of the muscarinic (M) receptor in the neuromuscular system of parasitic nematodes, which represents a special challenge for further research.

### 3.4 Effect of carveol on *Ascaris suum* nAChR expressed in *Xenopus* oocytes

**FIGURE 4:**
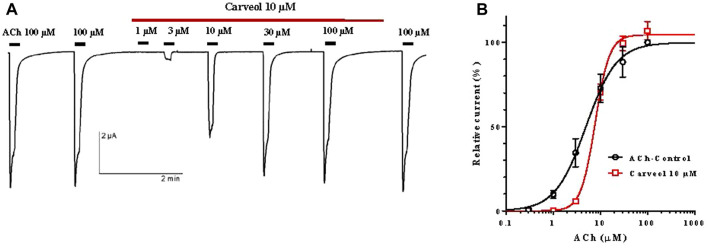
Carveol effect on the acetylcholine concentration-response relationships for the *Ascaris suum* ACR-16 nAChR expressed in *Xenopus* oocytes. **(A)** Recordings of ACh-evoked currents in presence of carveol 10 μM; **(B)** Concentration-response curves (non-linear regression) for acetylcholine control and in the presence of carveol 10 µM.

Following the results obtained on the *A. suum* neuromuscular preparations, we hypothesized that the effect of carveol 10 µM could be mediated through the nicotine-sensitive acetylcholine receptors (nAChR) of *A. suum*. In order to assess carveol at the receptor level, we expressed the *A. suum* nAChR in *Xenopus laevis* oocytes and carried out electrophysiological studies as recently achieved for carvacrol (Trailovic et al., 2021). Indeed, the *A. suum* ACR-16 subunit has been shown to give rise to a robust functional homomeric nAChR in the *Xenopus* oocyte heterologous expression system (Abongwa et al., 2016). To check for the functional expression of the nAChRs in *Xenopus* oocytes micro-injected with Asu-ACR-16 cRNAs, acetylcholine 100 µM was first applied, leading to the recording of large currents in the µA range (Figure 4A). The application of ACh 100 µM was repeated a second time, giving a similar current amplitude. When carveol 10 µM (same concentration used in neuromuscular contraction experiments) was perfused in the recording chamber, we observed no direct agonistic action on the nAChR (Figure 4A). To investigate a possible antagonistic effect, the nAChRs were challenged by increasing concentrations of ACh ranging from 1 to 100 μM, without carveol or in the continued presence of carveol 10 µM. With current amplitudes normalized to the maximal response to 100 μM, we found that the ACh EC_50_ value of *A. suum* nAChR was 4.9 ± 1.2 µM (*n* = 11) in the absence of carveol (Figure 4B). In the presence of carveol 10 μM, the ACh concentration–response curve was characterized by an EC_50_ of 7.8 ± 1.1 µM (*n* = 4) which was not significantly different from the ACh EC_50_ value obtained without carveol (*p* = 0.077). Interestingly, the maximal response amplitude elicited by ACh 100 µM was increased by 7% in the presence of carveol 10 µM (*p* = 0.142) and continued to increase by 8.75% after removal of carveol (wash). Thus, carveol 10 µM non-significantly increased the ACh efficacy for the homomeric ACR16 *A. suum* nAChR*,* what could be one of the possible mechanisms accounting for the potentiating effect of ACh-induced contractions.

### 3.5 Investigation on rat isolated ileum

**FIGURE 5:**
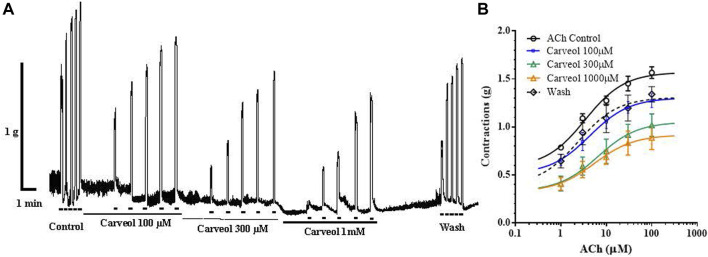
**(A)** Original recording of isometric contractions of isolated rat ileum induded by increasing concentrations of ACh and the effect of carveol on those contractions. *Short bar* indicates the application of ACh (1, 3, 10, 30 and 100 µM) *full line* the presence of carveol 100 μM; *dashed line* the presence of carveol 300 μM; *double full line* the presence of carveol 1 mM. **(B)** The concentration-response plot (non-linear regression) for ACh (*n* = 7), in the presence of carveol 100, 300 and 1,000 µM (*n* = 7) (mean ± S.E).

Carveol showed qualitatively different pharmacological effects on the neuromuscular system of nematodes compared to carvacrol in our earlier studies. In order to further analyze pharmacological characteristics of carveol, we tested its effects on contractions of the isolated rat ileum (Figure 5A). Increasing concentrations of ACh caused dose-dependent contractions of the ileum, where the EC_50_ was 6.51 ± 2.75 μM. Carveol applied in concentrations of 100, 300 and 1000 μM did not significantly affect the EC_50_ value of ACh, 4.31 ± 1.94, 6.51 ± 2.75 and 5.49 ± 3.1 μM (Figure 5B). However, incubation of ileum preparations with carveol caused a decrease of maximal contractile response to ACh. The control E_max_ was 1.57 ± 0.05 g, after incubation with 100 μM carveol it decreased to 1.30 ± 0.07 g, while after 300 and 1000 μM of carveol the E_max_ significantly further decreased to 1.05 ± 0.12 g (*p* = 0.001) and 0.91 ± 0.10 g (*p* < 0.0001), respectively. This inhibitory effect on ACh-induced contractions was reversible and after washing E_max_ increased to the control level, 1.31 ± 0.10 g (Figures 5A, B).

**FIGURE 6:**
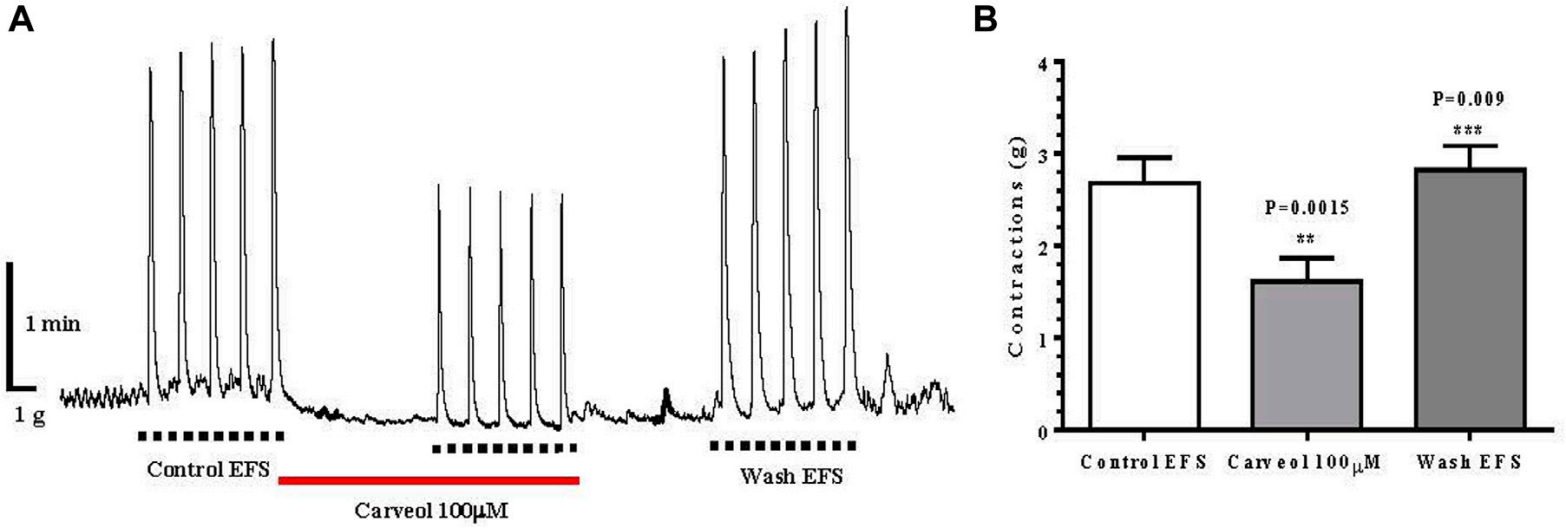
**(A)** Original recording of isometric contractions of isolated rat ileum indued by electrical fild stimulation every 60 s, package of 5 (EFS: 50 Hz, for 2 s, 1.0 ms pulse duration, 50 V voltage); *full line* the presence of carveol 100 μM; **(B)** Graphic presentation of mean values of contractions (ANOVA, *n* = 5) (mean ± S.E).

In addition to the effect of carveol on contractions of the isolated ileum induced by ACh, we also examined the effect of this monoterpenoid on the contractions of the ileum induced by EFS. The mean value of control contractions was 2.70 ± 0.27 g, while the incubation of the preparation with carveol 100 μM resulted in a significant inhibition of contractions, with a mean value of 1.60 ± 0.25 g (ANOVA *p* = 0.0015). Furthermore, this inhibitory effect of carveol was reversible and after washing it exceeded the control value, which was 2.80 ± 0.26 g (ANOVA *p* = 0.009) (Figures 6A, B).

**FIGURE 7:**
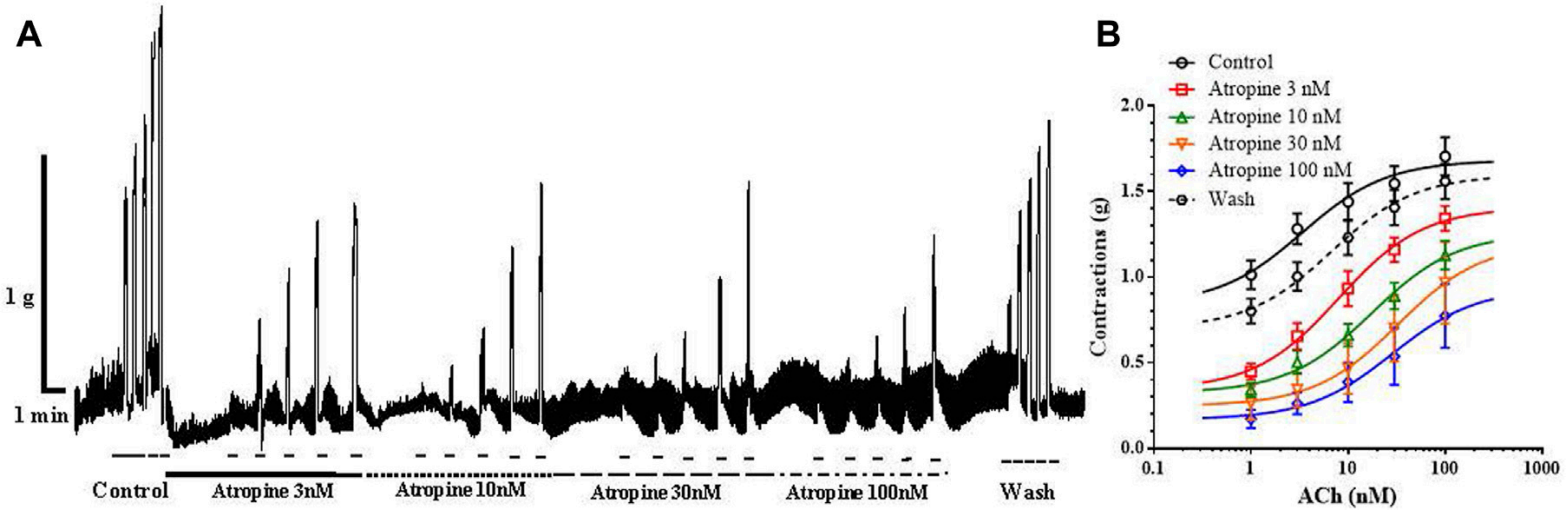
**(A)** Original recording of isometric contractions of isolated rat ileum indued by increasing concentrations of ACh and the effect of atropine on those contractions. *Short bar* indicates the application of ACh (1, 3, 10, 30 and 100 µM), *full line* the presence of atropine 3 nM; *dashed line* the presence of atropine 10 nM; *long dashed line* the presence of atropine 30 µM and *dash dot line* the presence of atropine 100 nM. **(B)** The concentration-response plot (non-linear regression) for ACh (*n* = 7), in the presence of atropine 3, 10, 30 and 100 nM (*n* = 7) (mean ± S.E).

In the next series of experiments, we tested the effect of increasing concentrations of atropine on ACh-induced ileal contractions, in order to compare the result with the previously observed inhibitory effect of carveol. The control EC_50_ of ACh was 3.75 ± 2.50μM, while in the presence of atropine 3, 10, 30 and 100 nM this value significantly increased to 7.99 ± 1.62 μM, 17.61 ± 1.65 μM (*p* = 0.0001), 32.85 ± 1.77 μM (*p* = 0.0001) and 28.73 ± 1.69 μM (*p* = 0.0001). On the other hand, incubation of preparations with atropine 3, 10 and 30 nM reduced the E_max_ of ACh from the control 1.68 ± 0.09 g to 1.41 ± 0.09 g, 1.26 ± 0.12 g, 1.20 ± 0.18 g, but only the highest tested concentration (100 nM) caused a significant reduction, it was 0.93 ± 0.13 g (*p* = 0.0004) (Figures 7A, B). The effect of atropine was reversible and after washing the EC_50_ and E_max_ of ACh returned to the control level (6.71 ± 1.99nM and 1.60 ± 0.11 g).

### 3.6 Investigation on rat isolated diaphragm

**FIGURE 8:**
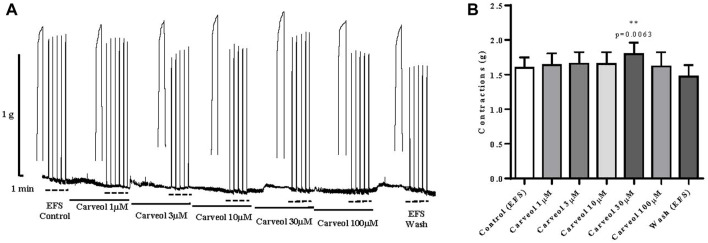
**(A)** Original recording of rat diaphragm contractions induced by EFS and the effects of increasing concentrations of carveol (1µM, 3µM, 104µM, 30µM and 100 µM) on contractions; **(B)** Graphic presentation of mean values of contractions (ANOVA; *n* = 7).

Finally, the effect of carveol on a skeletal muscle model, i.e. neuromuscular preparation of rat diaphragm was tested. Contractions were induced by EFS, and preparations were incubated in increasing concentrations of carveol 1, 3, 10, 30 and 100 μM (Figure 8A). Carveol caused a slight increase in amplitude of contractions from control 1.60 ± 0.14 g to 1.64 ± 0.17 g, 1.66 ± 0.17 g and 1.65 ± 0.17 g. Carveol concentration of 30 μM caused a significant increase of the contraction’s amplitude to 1.80 ± 0.17 g (*p* = 0.0063). On the other hand, 100 μM of carveol led to a reduction of the amplitude back to the control level, 1.62 ± 0.21 g (Figures 8A,B).

**FIGURE 9:**
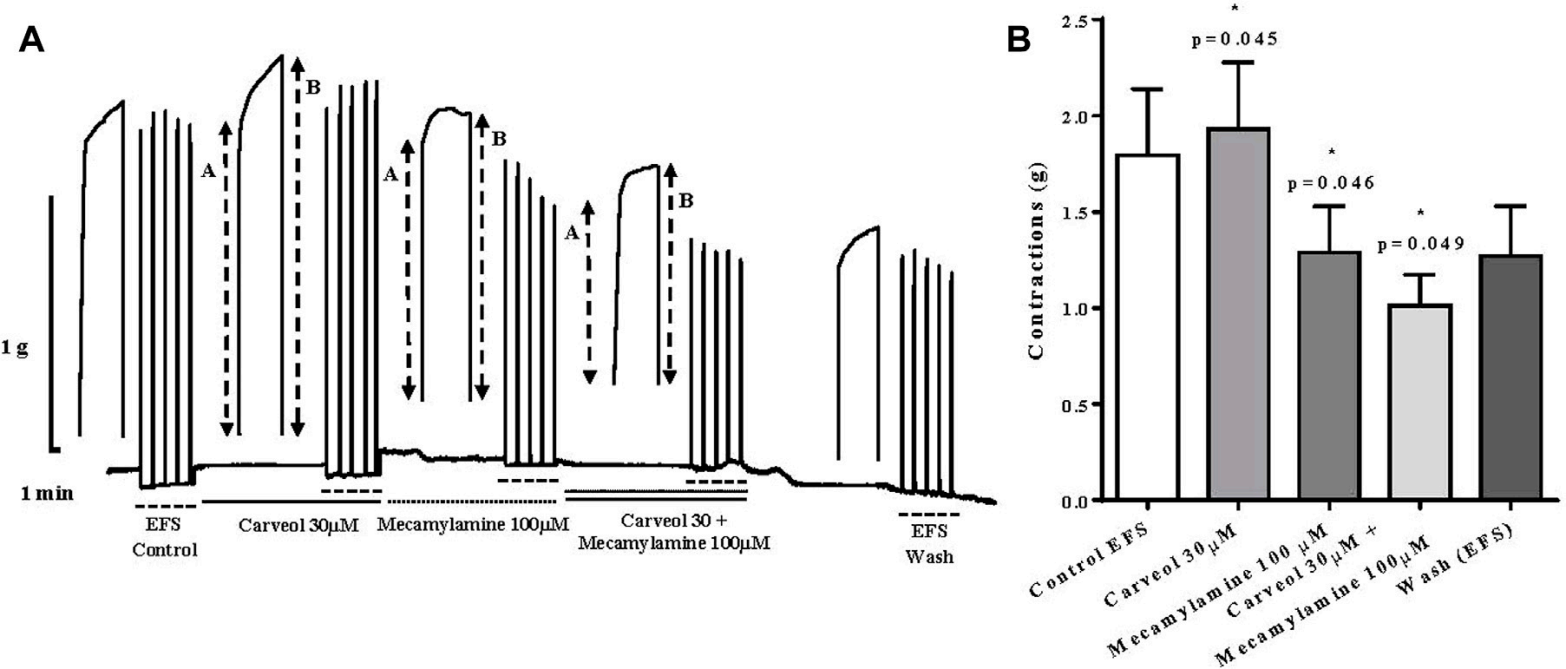
**(A)** Original recording of rat diaphragm contractions induced by EFS and the effects of carveol (30 μM) and mecamylamine (100 μM) on contractions, A-initial tetanic tension at the beginning of the tetanic stimulus, B- the tension at the end of the tetanic stimulus; **(B)** Graphic presentation of mean values of contractions (ANOVA, *n* = 5).

In the next series of experiments, we compared the effect of 30 μM of carveol and 100 μM of mecamylamine, a non-competitive nicotinic receptor antagonist. Carveol 30 μM significantly increased contractions of the isolated diaphragm induced by EFS, from control 1.79 ± 0.34g to 1.93 ± 0.34 g (*p* = 0.045). On the other hand, compared to the control, mecamylamine significantly reduced the amplitude of contractions to 1.29 ± 0.23 g (*p* = 0.046). When the diaphragm preparations were incubated simultaneously with 30 μM of carveol and 100 μM of mecamylamine, the inhibition of contractions was even more pronounced and the mean amplitude was 1.01 ± 0.16 g (*p* = 0.049) (Figures 9A, B).

We emphasize that the ratio (R) between the tension at the end (B) and the maximum tension at the beginning of individual tetanic contractions (A) was also analyzed (R = A/B). Increasing concentrations of carveol (1, 3, 10 and 30 μM), in addition to the potentiation of contractions, also led to an increase in the R value. However, carveol 100 μM, in addition to reducing the amplitude, also reduced the R value (Figure 9A). Furthermore, in the next series of contractions, carveol 30 μM increased the amplitude of contractions as well as the R value, while mecamylamine led to inhibition of the contraction amplitude and caused a tetanic fade. When we used carveol at the same time with mecamylamine, there was no tetanic fade, but the inhibition of the amplitude of contractions was more pronounced. This effect was reversible and contractions slightly increased after washing (Figure 9A).

## 4 Discussion

Molecular docking analysis indicated potential differences in the binding of carvacrol, geraniol and carveol to ACR-16, a homomeric nAChR widely distributed in *Ascaris* tissues. This is primarily related to the different geometry of the carveol binding site compared to carvacrol and geraniol. Carveol most likely binds to two different sites, one in the alpha and one in the beta domain of the receptor subunit. This could mean that carveol may exert different effects after binding to the receptor. Also, carveol shows a higher affinity for the orthosteric binding site compared to the allosteric site. On the other hand, geraniol can bind to two sites in the alpha domain, and its presence, or the presence of carvacrol, enhances their binding to the receptor. It was previously shown that carvacrol dominantly exhibited characteristics of a non-competitive antagonist of nAChR in *A. suum* (Marjanović et al., 2021; Trailovic et al., 2021). We verified this prediction of molecular interaction by examining the effects of carveol on the motility and survival of *C. elegans*. In our study carveol did not show a distinct time-dependent effect, but obtained LC_50_ value similar to published results for carvacrol and thymol (Lei et al., 2010). It is also interesting that carveol caused spastic paralysis of worms.

Our prediction about a qualitatively different effect of carveol on the neuromuscular system of nematodes compared to carvacrol proved to be correct. Unlike carvacrol, carveol significantly potentiated the contractile effect of ACh, reducing its EC_50_ by 58% and increasing the maximal contractile response (E_max_) by 10%. Quite differently, carvacrol exhibits non-competitive inhibition of ACh-induced contractions of *A. suum* neuromuscular preparations (Trailović et al., 2015; Marjanović et al., 2021). Besides, the E_max_ continued to increase even after removal of carveol, which we also previously saw with the effect of neuropeptide AF2 in *A. suum* (Trailovic et al., 2005). The fact that carveol significantly potentiated the effect of ACh by reducing its EC_50_ value and shifting the dose-response curve to the left, as well increasing the E_max_, confirmed our prediction that it binds mostly to the orthosteric site on nAChR subunit. In order to investigate the mode of action of carveol, we applied carveol 10 µM on *Xenopus* oocytes expressing the *A. suum* nAChR and observed neither agonistic, nor antagonistic effect using the two-electrode voltage-clamp electrophysiology approach. Nevertheless, carveol increased the efficacy of ACh activation and, surprisingly, the ACh maximal response amplitude continued to increase even after removal of carveol. This result confirmed that part of the potentiating effect of carveol on ACh-induced contractions of *A. suum* could be mediated by the nAChR. Furthermore, this potentiating effect on ACh-induced contractions exhibited by carveol were partially sensitive to atropine. Atropine increased the EC_50_ in the presence of carveol by about 15% and decreased the E_max_ by 10%. Interestingly, atropine only partially inhibited *A. suum* muscle cell membrane depolarization induced by the muscarinic agonist 5-methylfurmethiodide (Trailović et al., 2008). Obviously, there is still some non-nicotine target to which carveol can bind and further enhance contractions.

Unfortunately, there is not much information in literature about the effect of carveol on parasitic nematodes of humans and animals. Most of the published researches refer to the antinematodal effects of essential plant oils which, among other ingredients, have these two monoterpenoids in their composition. Also, that research mostly refers to plant-parasitic root-knot nematodes (Echeverrigaray et al., 2010).

To compare the pharmacological effects of carveol in invertebrates (potential parasites) and mammals (hosts), we tested its effect on contractions of the isolated rat ileum induced by increasing concentrations of ACh. Carveol in concentrations of 100, 300 and 1000 μM did not significantly change the value of EC_50_ for ACh, but significantly reduced E_max_ of contractions for 18, 33% and 42%. Also, carveol at a concentration of 100 μM significantly inhibits EFS-induced contractions, by 41%. This inhibitory effect of carveol is reversible in both experiments and disappears after washing. ACh is released from cholinergic nervous terminals in the myenteric plexus, resulting in contraction of the intestinal smooth muscle through activation of M receptors (Furness, 2009). Primarily M_3_ but also the M_2_ receptor, play the main role in the contraction of the ileal smooth muscles (Unno et al., 2005; Takeuchi et al., 2007). To check the similarities/differences in the inhibitory effect on ACh-induced ileal contractions, we tested the effect of increasing concentrations of atropine, the non-selective muscarinic acetylcholine receptor (mAChR) antagonist. Unlike carveol, increasing concentrations of atropine significantly increased the EC_50_ of ACh, and decreased the E_max,_ but only significant in the highest tested concentration (100 nM). This is a different effect compared to the effect of carveol because carveol exerts the effect of a non-competitive antagonist. Our results are in agreement with data published by Zholos and Bolton, (1997). This would mean that atropine in lower concentrations binds M2 receptors and increases the EC_50_ value, while in higher concentrations more intensively activated the M3 receptors, which results in a decrease in E_max_. On the other hand, it is obvious that if carveol has ability to block M receptors in ileum acting probably predominantly on the M3 type.

In the last part of the study, we tested the effect of carveol on EFS-induced contractions of the isolated rat diaphragm. It was shown that increasing concentrations of carveol slightly increase the amplitude of contractions, as 30 μM caused a significant increase, along with an increase in R values. However, the next higher tested concentration of 100 μM led to inhibition of contractions and return to the control level, with a decrease in the R value. In order to analyze the effect of carveol on the neuromuscular synapse in more detail, we compared the effect of mecamylamine 100μM, a nicotinic receptor antagonist, and tested the effect of carveol 30 μM. As before, carveol again significantly increased the amplitude of EFS-induced contractions as well as R value, while mecamylamine significantly reduced contractions and caused a tetanic fade. However, the simultaneous incubation of the diaphragm with carveol and mecamylamine showed an even stronger inhibitory effect on the amplitude of contractions, but there was no tetanic fade. Mecamylamine (0.1–300 μM) is able to produce a very intense fade of tetanic contractions simultaneously with a significant reduction of the maximal tetanic tension (Faria et al., 2003). The reason is the mecamylamine’s ability to block pre- and postsynaptic nAChR at the neuromuscular synapse. Mecamylamine is a non-selective nAChR antagonist. It effectively acts on the muscle-type of postsynaptic nAChR as well as on the α3β2 facilitatory presynaptic autoreceptor at the neuromuscular junction (NMJ). In the presented study, carveol exhibited a specific effect on EFS-induced contractions of the diaphragm. Carveol was potentiating contractions up to a concentration of 30 μM and then inhibiting the amplitude of contractions at a higher concentration (100 μM), but without causing a tetanic fade. We can interpret this effect as an effect of partial agonist/antagonist on the postsynaptic nAChR, but the question of the presynaptic effect remains. Namely, in combination with mecamylamine, carveol increased its inhibitory effect but neutralized the effect of the tetanic fade. There are at least two possible explanations for this effect of carveol. The first possibility is that carveol could competitively stimulate the presynaptic nACh autoreceptor at neuromuscular junction (NMJ) with higher affinity and overrides the inhibitory effect of mecamylamine. There is at least one other possibility, which stems from the previously observed effect of carveol on the *A. suum* neuromuscular synapse. Namely, we saw that atropine partially reduced the potentiating effect of carveol on ACh-induced contractions in *A. suum*. It is possible that carveol neutralizes tetanic fades by inhibition the presynaptic M_1_ autoreceptor, which is not affected by mecamylamine.

## 5 Conclusion

Presented research has shown that carveol in nematodes potentiates contractions caused by ACh and that this effect could be mediated by the nAChR. Further research should provide an answer how this effect can be used to potentiate the effectiveness of the antinematodal drugs which are nAChR agonists in nematodes (imidazothiazoles and tetrahydropyrimidines). On the other hand, carveol exhibited specific pharmacological effects on mammalian neuromuscular preparations. In the ileum, carveol acts as a non-competitive antagonist, reducing the intensity of contractions but not affecting EC_50_ of ACh. Furthermore, in the skeletal muscle model, carveol achieves a qualitatively different effect on the presynaptic and postsynaptic nACh receptors, which also requires further investigations, not only on the nature of synaptic regulation, but also for the potential practical therapeutical application of carveol in neuromuscular diseases.

Based on the obtained results, it can be considered that carveol represents a promising platform for the development of a potentially new class of anthelmintics and prospective tools for the studies of neuromuscular neurotransmission.

## Data Availability

The datasets presented in this study can be found in online repositories. The names of the repository/repositories and accession number(s) can be found in the article/Supplementary Material.
