# Corrigendum: Pharmacological effects of monoterpene carveol on the neuromuscular system of nematodes and mammals

**DOI:** 10.3389/fphar.2024.1466575

**Published:** 2024-08-09

**Authors:** Maja Stojković, Zoran Todorović, Dragana Protic, Strahinja Stevanovic, Dragana Medić, Claude L. Charvet, Elise Courtot, Djordje S. Marjanović, Jelena Nedeljković Trailović, Saša M. Trailović

**Affiliations:** ^1^ Department of Pharmacology, Clinical Pharmacology and Toxicology, Faculty of Medicine, University of Belgrade, Belgrade, Serbia; ^2^ PR CYNNAB, Belgrade, Serbia; ^3^ Department of Pharmacology and Toxicology, Faculty of Veterinary Medicine, University of Belgrade, Belgrade, Serbia; ^4^ INRAE, Université de Tours, ISPF-37380, Nouzilly, France; ^5^ Department of Nutrition and Botany, Faculty of Veterinary Medicine, University of Belgrade, Belgrade, Serbia

**Keywords:** carveol, C. *elegans*, *Xenopus* oocytes, A. *suum*, AChR

In the published article, there was an error in the **Author** list, and author Elise Courtot was erroneously excluded. The corrected author list appears below.

“Maja Stojković^1^, Zoran Todorović^1^, Dragana Protić^1^, Strahinja Stevanovic^2^, Dragana Medić^3^, Claude L. Charvet^4^, Elise Courtot^4^, Djordje S. Marjanović^3^, Jelena Nedeljković Trailović^5^, Saša M. Trailović^3,^*”

The authors apologize for this error and state that this does not change the scientific conclusions of the article in any way. The original article has been updated.

