# Supplementary material for: Pharmacological effects of monoterpene carveol on the neuromuscular system of nematodes and mammals
Source: Front Pharmacol. 2024 Jan 22;15:1326779. doi: 10.3389/fphar.2024.1326779 (PMC10839021; doi:10.3389/fphar.2024.1326779)
Supplement: Supplementary file 2 [file DataSheet1.DOCX]

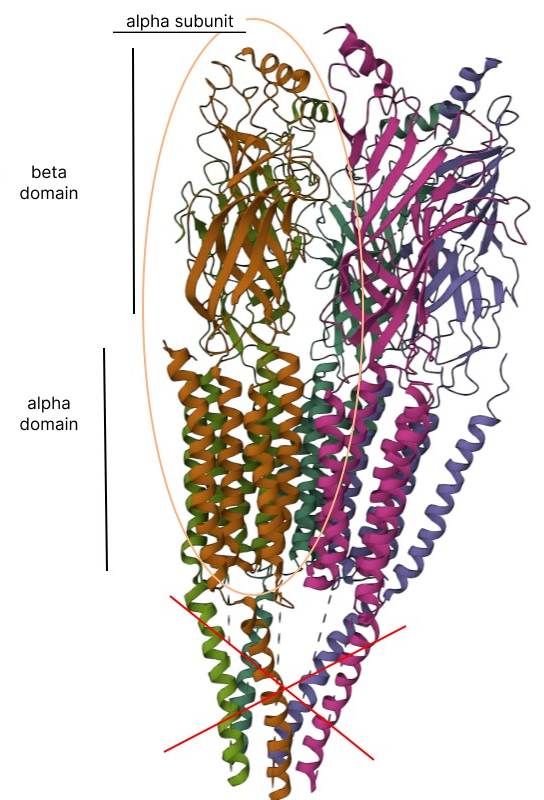


**Figure S1.** Refined structure of nicotinic acetylcholine receptor, *T. marmarota.* PDB structure contains 5 chains (A-E) of which A and D are identical, annotated alpha subunit used as a template for ACR-16 model.
